# GC/MS analysis of hypoxic volatile metabolic markers in the MDA-MB-231 breast cancer cell line

**DOI:** 10.3389/fmolb.2023.1178269

**Published:** 2023-05-11

**Authors:** Theo Issitt, Matthew Reilly, Sean T. Sweeney, William J. Brackenbury, Kelly R. Redeker

**Affiliations:** ^1^ Department of Biology, University of York, York, United Kingdom; ^2^ York Biomedical Research Institute, University of York, York, United Kingdom

**Keywords:** hypoxia, VOC, cancer, breast cancer, volatile flux, hypoxic, GC/MS, metabolism

## Abstract

Hypoxia in disease describes persistent low oxygen conditions, observed in a range of pathologies, including cancer. In the discovery of biomarkers in biological models, pathophysiological traits present a source of translatable metabolic products for the diagnosis of disease in humans. Part of the metabolome is represented by its volatile, gaseous fraction; the volatilome. Human volatile profiles, such as those found in breath, are able to diagnose disease, however accurate volatile biomarker discovery is required to target reliable biomarkers to develop new diagnostic tools. Using custom chambers to control oxygen levels and facilitate headspace sampling, the MDA-MB-231 breast cancer cell line was exposed to hypoxia (1% oxygen) for 24 h. The maintenance of hypoxic conditions in the system was successfully validated over this time period. Targeted and untargeted gas chromatography mass spectrometry approaches revealed four significantly altered volatile organic compounds when compared to control cells. Three compounds were actively consumed by cells: methyl chloride, acetone and n-Hexane. Cells under hypoxia also produced significant amounts of styrene. This work presents a novel methodology for identification of volatile metabolisms under controlled gas conditions with novel observations of volatile metabolisms by breast cancer cells.

## 1 Introduction

The human “volatilome” describes the production and metabolism by the human body of small, carbon-containing compounds called volatile organic compounds (VOCs) which are gaseous at room temperature and pressure (Amann et al., 2014; Drabinska et al., 2021). VOCs can be found in abundance in the breath and are reflective of processes within the body (Drabinska et al., 2021; Issitt et al., 2022a). Although fluctuations of VOCs vary between individuals and throughout the day, disease specific “volatile fluxes,” or biomarkers, could provide opportunities to non-invasively diagnose disease, monitor treatment and measure bodily functions (Issitt et al., 2022a; Issitt et al., 2022b).

The clinical potential of VOCs in diagnosis has been shown by a number of published breath studies (Issitt et al., 2022a). Diagnostic accuracy using breath VOC biomarkers has been achieved for a wide range of conditions, including various types of cancer (Issitt et al., 2022a; Jia et al., 2019), liver disease (De Vincentis et al., 2019), diabetes (Das et al., 2016), transplant rejection (Phillips et al., 2004), infections of the lung (Issitt et al., 2022a; Beccaria et al., 2018), liver function (using labelled VOCs) (Sangnes et al., 2019) and other conditions (Issitt et al., 2022a). Each study may independently achieve high sensitivity of disease detection (i.e., >90%) but the reported compounds often do not translate between studies, slowing clinical application through conflicting and confounding results (Issitt et al., 2022a). However, our recent meta-analysis has shown underlying trends in chemical functional groups from published studies supporting potential clinical application (Issitt et al., 2022a). It is clear that in order to identify effective biomarkers more targeted methodological approaches are required to overcome variability (Issitt et al., 2022a; Hanna et al., 2019).

VOC profiles from cell types associated with pathological conditions have been identified, for example, differences between breast (Issitt et al., 2022b; Lavra et al., 2015), liver (Mochalski et al., 2013) and mesothelioma (Little et al., 2020) cancer cell lines. However, cellular VOC studies tend to be non-stressed cells in high (21%, atmospheric) oxygen conditions, which is not consistent with many disease or normal physiological states. To accelerate biomarker discovery, we propose models of pathophysiological stress. For example; stress from reactive oxygen species (ROS) induces alkane release in breast cancer cells (Liu et al., 2019), VOCs which have been observed in the breath of ROS associated conditions (Issitt et al., 2022a).

Hypoxia is a persistent reduction in oxygen from normal physiological conditions (normoxia). It is characteristic of a range of diseases, including, pulmonary hypertension (Young et al., 2019) and cancer (SamantaandSemenza, 2018). It induces a range of metabolic alterations, including reduction in adenosine triphosphate generation and inhibition of fatty-acid desaturation through hypoxia inducible factor activity (WheatonandChandel, 2011; SamantaandSemenza, 2018; Young et al., 2019), which can produce alterations in a range of associated breath volatiles (Harshman et al., 2015; Mazzatenta et al., 2021). Despite its relevance to pathophysiology, hypoxic volatiles have yet to be investigated *in vitro*. This is partially due to the challenges associated with development of a headspace sampling tool which can maintain an hypoxic environment. While volatile compounds in the available, limited, published studies associated with hypoxia show variation in breath (Harshman et al., 2015; Mazzatenta et al., 2021), translatable studies are required for target biomarker discovery.

Biomarker discovery in appropriate biological models can accelerate clinical delivery by identifying and allowing targeted analytical approaches, separating methodical challenges from pathology, and improving sensitivity. Multi-timepoint sampling and approaches considering local environment will also accelerate clinical application of breath diagnostics and consideration of methodological challenges around clinical application should drive experimental design. We have previously demonstrated a platform and method for both identification of VOC metabolisms in cellular headspace over time and VOC changes in response to cellular stress (Issitt et al., 2022b). However, models of pathological conditions require further investigation to ensure biomarker discovery is translatable from cell to human.

One of the primary sources of variance within the published literature revolves around methodology. Methods of breath VOC analysis can be split into 3 main sections where variability between studies can arise: initial collection, sample transfer and analytical approach. There are many effective breath collection methods for analysis of VOCs, such as simply breathing into a specialised bag or use of specialised technologies (Hanna et al., 2019; Di Gilio et al., 2020). Many studies use single time point collection (Issitt et al., 2022a), considering presence verses absence, which can miss valuable metabolic information, particularly volatile uptake, driven via chemical reactions reflective of cellular state or through cellular metabolism. Furthermore, variability in local environment influences and reduces reported outcome precision (Issitt et al., 2022a; Di Gilio et al., 2020; Doran et al., 2017) and approaches should consider sampling the environment (i.e., ambient air) along with breath (Hanna et al., 2019). A sample, once collected, is then transferred, either directly or indirectly (such as through chemical traps) to an analytical instrument. There are two main analytical approaches for discovery and accurate detection of VOCs: targeted and untargeted. Utargeted approaches, investigating the breath of patients, are capable of identifying relatively concentrated material (ppbv) whereas targeted approaches generally are capable of quantifying lower concentrations (pptv). Untargeted approaches therefore may miss changes in important, low-concentration compounds, while targeted approaches can only look only for a limited number of known compounds of interest, reducing discovery potential.

Here, hypoxic stress is applied to a well-studied breast cancer cell line with the intent of identifying process and disease-linked physiological volatile metabolisms specifically linked to low oxygen conditions, so that more accurate diagnostic tools can be developed and applied in the clinic. Both targeted and untargeted analyses are applied after sampling with a static headspace method that accounts for the ambient air background and allows quantification of cellular uptake of VOCs. It was predicted that upon successful maintenance of a hypoxic environment, cellular VOC profiles from hypoxic versus hyperoxic cellular models would alter significantly.

## 2 Methods

Methods for culture of MDA-MB-231 cells, headspace sampling from custom chambers and GC/MS analysis have been previously described in detail (Issitt et al., 2022b).

### 2.1 Cell culture

MDA-MB-231 breast cancer cells (a gift from Professor Mustafa Djamgoz, Imperial College London) were grown in Dulbecco’s Modified Eagle Medium (DMEM, Thermo Scientific, Waltham, MA, United States), 25 mM glucose, supplemented with L-glutamine (4 mM) and 5% foetal bovine serum (Thermo Scientific, Waltham, MA, United States). Cell culture medium was supplemented with 0.1 mM NaI and 1 mM NaBr (to model physiological availability of iodine and bromide). All cells were grown at 37°C with 5% CO_2_.

Prior to volatile collection, cells were trypsinised, and 500,000 cells were seeded into 8 mL complete media in 10 cm polystyrene cell culture dishes. Cells were then allowed to attach for 3-4 h, washed with warm PBS and 6 mL treatment media was applied. Volatile headspace sampling was performed 24 h later.

### 2.2 Induction of the hypoxic environment and VOC headspace sampling

Cells were placed in static headspace chambers as previously described (Issitt et al., 2022b) with new, clean silicon gaskets. Low oxygen, hypoxic gas (1% O_2_, 5% CO_2_, and 94% N_2_; purchased from BOC Specialty Gases, Woking, United Kingdom) was flushed through the chambers at a rate of 4 L/min for 10 min (chamber volume = 25 L). Chambers were then closed and placed at 37°C for 2 h to allow residual oxygen in the media to equilibrate with chamber headspace. Chambers were then flushed again at a rate of 4 L/min for 10 min, sealed and returned to 37°C.

After a further 24 h, chambers were flushed again at a rate of 4 L/min for 10 min 15 mL of gas standards (MeCl, 520 ppb (parts per billion); MeBr, 22 ppb; MeI, 26 ppb; DMS, 110 ppb; CFC-11, 400 ppb and CHCl_3_, 110 ppb; BOC Specialty Gases, Woking, UK) were then injected into the chambers through a butyl seal and time zero sample taken. Injected compounds are either known metabolites for cancer cells, or internal standards (CFC-11) for the analysis and quantification of metabolism. Final chamber concentrations were similar to environmental concentrations, e.g., MeCl, 1.2 ppb and MeBr 0.05 ppb, particularly more polluted urban spaces (Redeker et al., 2007). Injected gases are the same as those used for calibration. Compounds not injected but detected at first time point, due to residual presence from laboratory air, (including isoprene, acetone, 2-MP, 3-MP and n-hexane) were quantified. Two time zero (T0) samples were taken using an evacuated 500 mL electropolished stainless steel canister (LabCommerce, San Jose, United States) through fine mesh Ascarite® traps (Archbold et al., 2005), after which the chamber was resealed and left on a platform rocker on its slowest setting for 120 min, at which point two further air samples (T1) were collected. Duplicate samples were analysed with targeted and untargeted MS approaches.

Cells were removed from the chamber, washed with PBS twice and lysed in 500 µL RIPA buffer (NaCl, 5 M; 5 mL Tris-HCl, 1 M, pH 8.0; 1 mL Nonidet P-40; 5 mL sodium deoxycholate, 10%; 1 mL SDS, 10%) with protease inhibitor (Sigma-Aldrich, Roche; Mannheim, Germany). Protein concentration of lysates were determined using BCA assay (Thermo Scientific, Waltham, MA, United States).

Media alone was taken through exactly the same process as cells. This has been visualised in Supplementary Figure S1. Only acetone was shown to have any significant variability between conditions. These media blank outcome averages were subtracted from respective cellular samples prior to protein normalisation. Comparative controls include lab air blanks and those data available from the dataset and collection method published previously which created and quantified metabolic fluxes of volatile compounds from MDA-MB-231 under hyperoxic (lab air) conditions (Issitt et al., 2022b).

### 2.3 Sample collection and GC/HID analysis

Ten mL headspace samples were taken from chambers using an airtight syringe (10 mL, SGE, Trajan, Milton Keynes, UK). 1% O_2_ (BOC Specialty Gases, Woking, UK) was flushed through sealed chambers containing 6 mL DMEM as described for cell treatments. Samples were taken at 5 and then 10 min post initial flush. In order to replicate cell treatments, the chamber was then closed for 2 h, then flushed for 10 min, after which an air sample was taken. A further 20 min flush with 1% O_2_ air was employed and the chamber was closed, placed at 37°C, and left to incubate for 24 h, at which time the final sample was taken.

Air samples were immediately analyzed with a SRI 8610C Gas Chromatograph connected to a SRI 8690-0030 Helium Ionisation Detector (GC/HID (SRI Instruments Europe GmbH, Torrance, CA, United States). Peak separation was achieved using a Restek© PORAPAK Q porous polymer column (1.83 m × 2.1 mm ID × 3.175 mm OD), a solenoid switching valve (for backflushing CO_2_) and a Restek© MOLECULAR 5 A sieve column (0.91 m × 2.1 mm ID × 3.175 OD) (Restek©, Bellefonte, PN, United States) connected in series. Helium was used as a carrier gas at 18 psi, and the flow rate and column temperatures (50°C) were maintained during separation. The valve was switched at 1.5 min to backflush the PORAPAK Q column. Measurement of compounds eluted from the MOLECULAR 5 A sieve was achieved by using an SRI 8690-0030 Helium Ionisation Detector. SRI PeakSimple (version 453) software was used to generate a digital chromatograph for each sample and O_2_ was quantified by comparing the peak area to known standards.

The standard curve was developed by flushing 120 mL Wheaton vials with butyl stoppers with pure nitrogen (BOC Gases, Woking, UK) for 30 min. Ten mL of nitrogen only was injected to establish a background control. Because atmospheric air at sea level contains 21% O_2_, lab air was injected at 1%, 2%, 10%, 20%, and 30% within the N_2_-filled vial to generate a standard curve consisting of 0%, 0.21%, 0.42%, 2.1%, 4.2%, and 6.3% and 21% (lab air only). Peak areas were integrated using Graphpad (Prism), and Padé (1, 1). Linear regression demonstrated an R squared value of 0.96.

### 2.4 GC/MS analysis of VOCs

Collected canister samples were transferred to a liquid nitrogen trap through pressure differential. Pressure change between beginning and end of “injection” was measured, allowing calculation of the moles of canister collected air injected Sample in the trap was then transferred, via heated helium flow, to an Aglient/HP 5972 MSD system (Santa Clara, CA, United States) equipped with a PoraBond Q column (25 m × 0.32 mm × 0.5 μm film thickness) (Restek©, Bellefonte, PN, United States). Targeted samples were analyzed in selected ion monitoring (SIM) mode, and untargeted samples in full scan (SCAN) mode with the mass range of 45–200 amu. The mass spectrometer was operated in electron impact ionization mode with 70 eV ionization energy, and transfer line, ion source, and quadrupole temperatures of 250, 280, and 280, respectively. For details on SIM and significantly altered, identified SCAN compounds, see Table 1. All samples were analysed within 6 days of collection. The oven program for both SIM and SCAN analyses were identical and are as follows: 35°C for 2 min, 10°C/min to 155°C, 1°C/min to 131°C, and 25°C/min to 250 with a 5 min 30 s hold.

**TABLE 1:** Retention times, mass charge ratios and GC/MS modes used to characterise individual VOCs. SIM and SCAN refer to selected ion monitoring and full mass scanning (targeted and untargeted) GC/MS modes.

Compound	Retention time (min)	Mass charge ratio (m/z)
SIM		
Methy l chloride (MeCI)	7.6–7.9	50,52
Methy l bromide (MeBr)	10.3–10.4	94,96
Trichloroflouromethane (CFC-11)	15.0–15.3	101,103
Methy l iodide (Me l)	15.4–15.7	127,142
Dimethyl Sulfide (DMS)	16.2–16.5	62
acetone	18.2–18.4	58
lsoprene	18.4–18.6	Total ion count
Trichloromethane (CHCl3)	25.4–25.7	83,85
2-Methyl pentane (2-MP)	27.6–27.8	43,57
3-Methy lpentane (3-MP)	28.0–28.2	43,57
n-Hexane (n-Hex)	28.5–28.7	43,57
SCAN		
Styrene	33.3–33.5	45–200 amu

Calibration was performed using standard gases (BOC Specialty Gases, Woking, UK). Linear regression of calibration curves confirmed strong, positive linear relationships between observed compound peak areas and moles of gas injected for each VOC (*r* ^2^ > 0.9 in all cases). For compounds not purchased in gaseous state (BOC Specialty gases, as above), 1-2 mL of compound in liquid phase was injected neat into butyl sealed Wheaton-style glass vials (100 mL) and allowed to equilibrate for 1 h. One mL of headspace air was then removed from neat vial headspace using a gas tight syringe (Trajan, SGE) and injected into the headspace of a second 100 mL butyl sealed Wheaton-style glass vial. This was then repeated, and 1 mL of the 2nd serial dilution vial was injected into the GC/MS system with 29 mL of lab air to give ppb concentrations. This was performed for methanethiol (MeSH, SPEXorganics, St Neots, UK), isoprene (Alfa Aesar, Ward Hill, MA, United States), acetone (Sigma-Aldrich, Burlington, MA, United States), 2- & 3-methyl pentane and n-hexane (Thermo Scientific, Waltham, MA, United States). Reported compounds detected by the GC/MS were confirmed by matching retention times and mass–charge (*m*/*z*) ratios with known standards.

$$Equation\;1:\left[VOC\right]\left(ppt\right)=\frac{CF\;x\;10^{12}\;x\;Peak\;area\;x\;Calibration\;slope}{n}$$ (1)

Equation 1 outlines the approach to calculating VOC concentrations in parts-per-trillion-by-volume, or pptv. Here *Peak area* refers to the combined peak areas for the mass-charge ratios identified in Table 1. Multiplying *Peak areas* by their associated calibration curves (*Calibration Slope*) generate molar amounts which, when divided by the number of moles of headspace air injected (*n*), generate a unitless (moles compound/moles of air) ratio. Pptv concentrations are then obtained by multiplying this unitless ratio by 1 × 10^12^. For clarity, part-per-billion-by-volume values would be obtained by multiplying the unitless ratios by 1 × 10^9^, or one billion. Sample VOC concentrations were then normalised to CFC-11 concentrations [240 parts-per-trillion-by-volume (pptv)] through multiplication by a “correction factor,” or *CF*, Eq. 1). CFC-11 was used as an internal standard, since atmospheric concentrations of CFC-11 are globally consistent and stable (Redeker et al., 2007). Quantification of Styrene was done as above but normalisation to CFC-11 was not possible under flushed, hypoxic conditions.

To account for differences in rates of cellular proliferation over 24 h, cellular results from GC/MS analyses were normalised to protein content at time of sampling using a BCA assay. When comparing media blanks to cellular assays results are reported in grams compound per Petri dish per hour.

Data has been made publicly available at the National Institute of Health Metabolomics workbench (project PR001638, DOI: http://dx.doi.org/10.21228/M8ZX4D) (Sud et al., 2016).

### 2.5 Hydrogen peroxide (amplex red) assay

Experiments were performed in phenol red free DMEM. DMEM containing 50 μM Amplex Red reagent (Thermo Scientific, Waltham, MA, United States) and 0.1 U/mL horse radish peroxidase (HRP, Thermo Scientific, Waltham, MA, United States) was added to cells in 12 well dishes (500 μL per well) for 15 min following 24 h in hypoxic or control conditions. Fluorescence at 590 nm was measured with a plate reader (Clariostar, BMG, Ortenberg, Germany) and compared against a H_2_O_2_ standard curve for quantification.

### 2.6 Statistics

Figures were assembled and statically analysed in Graphpad Prism version 9.3. VOCs were separated based on their flux amount to allow visualisation on the *y*-axis and were analysed this way. Two-way ANOVA with Bonferroni post-hoc analysis was performed for graphs with multiple factors was performed (Figures 2A, B; Supplementary Figures S1A, B). One-way ANOVA with Tukey post-hoc analysis was performed for acetone analysis (Figure 2B; Supplementary Figure S1B). Student’s t-test was performed for Styrene analysis against media only as none was detected for control cells, and these were presented on the graph for visual information. Amplex red data was analysed using Student’s t-test.

## 3 Results

### 3.1 Chambers maintain low oxygen conditions over 24 h

**FIGURE 1:**
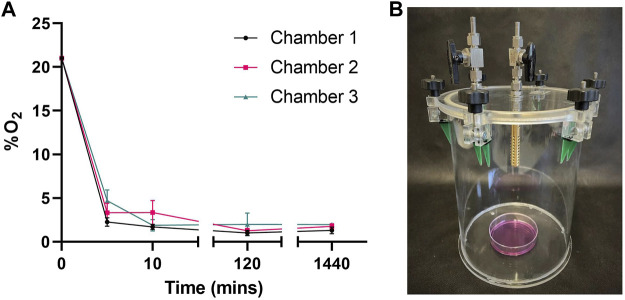
Chambers maintain hypoxic conditions over 24 h **(A)** Oxygen (O_2_) content in 3 custom made chambers containing 6 mL media was measured following a 10 min flush, 2 h dwell and another 10 min flush (20 mins) with 1% O_2_, 5% CO_2_ gas mix. O_2_°/o was then measured following chambers being sealed for 1440 min (24 h). Mean ± SEM; *n* = 3 **(B)** Image of collection chamber.

To confirm chambers maintained hypoxic conditions over 24 h we sampled gas from chambers throughout our method, measuring O_2_. When flushed with reduced oxygen air (1%) for 5 min, oxygen levels rapidly fell from atmospheric 21% to between 6% and 2% (Figure 1). After 10 min of reduced oxygen flushing, each chamber held less than 5%. Chambers left for 2 h (120 min) to allow media to equilibrate and flushed for 10 min revealed average O_2_ levels of 1.15% ± 1.03 (Ch 1), 1.34% ± 0.93 (Ch 2) and 1.98% ± 4.07 (Ch 3) respectively. Sealed chambers maintained low oxygen levels over 24 h with average O_2_ levels of 1.31% ± 1.31 (Ch 1), 1.76% ± 1.02 (Ch 2) and 1.96% ± 0.28 (Ch 3) respectively.

### 3.2 Hypoxia induces differing volatile fluxes in breast cancer cell line MDA-MB-231

**FIGURE 2:**
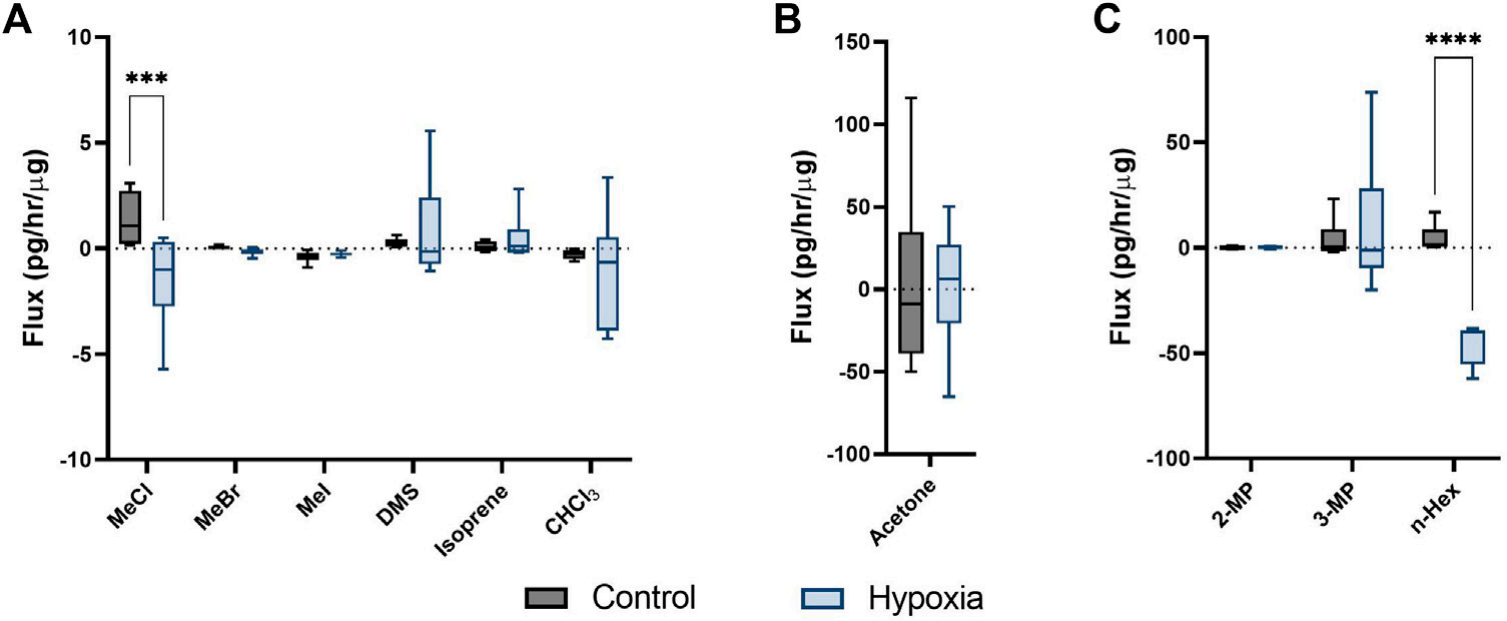
Cellular volatile response to hypoxia. Volatile flux (pg/hr/µg) for MDA-MB-231 cells in control conditions or hypoxia (24 h). Media subtracted and protein normalised VOC flux for MDA-MB-231 control cells (*n* = 6) and cells in hypoxia (*n* = 6). CHCl3, chloroform; OMS, dimethyl sulfide; MeBr, methyl bromide; MeCI, methyl chloride; Mel, methyl iodide; MeSH, methanoethiol; 2-MP, 2 methyl pentane; 3-MP, 3 methyl pentane; n-Hex, n-hexane. Boxplot whiskers show median ± Tukey distribution, *n* = 6. Two way ANOVA followed by Bonferroni post-hoc test was performed for **(A,B)**. One way ANOVA with Tukey post-hoc test performed for B; ***p < 0.001; ****p < 0.0001.

Persistent hypoxia over 24 h induced significant changes in flux for 3 targeted compounds (SIM analysis); MeCl, acetone and n-hexane (but not hexane isomers; 2-methyl pentane, or 3-methyl pentane), when compared to control (Figures 2A–C). MeCl was taken up by cells under hypoxia and released by cells under hyperoxic cell culture conditions. n-Hexane was produced by hyperoxic control cells while those under hypoxia consumed hexane.

### 3.3 Production of styrene under hypoxic conditions

**FIGURE 3:**
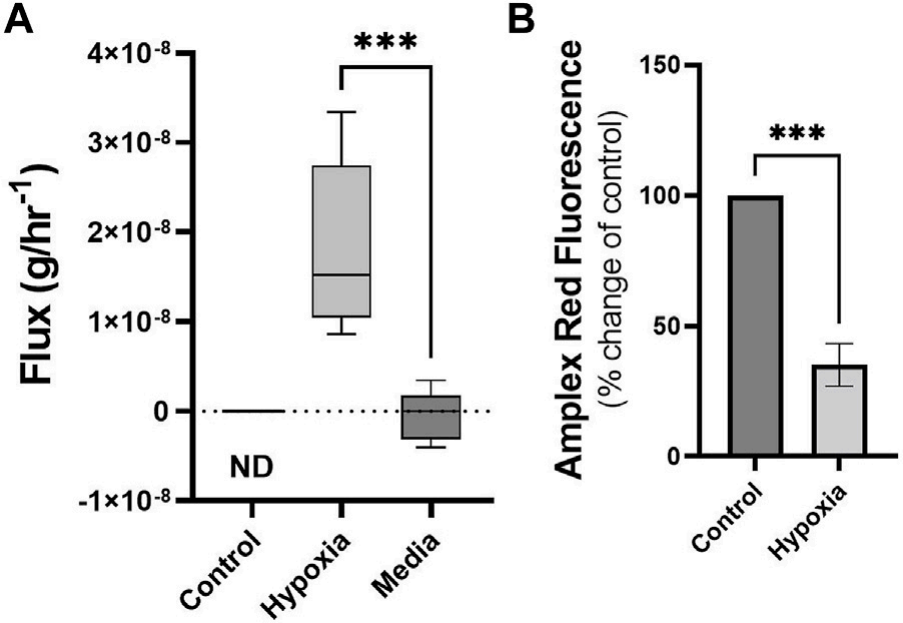
Cells under hypoxic conditions produce styrene and exhibit reduced ROS. Volatile flux (g/hr^−^1) for styrene from MDA-MB-231 cells in control conditions or hypoxia and media only (24 h). Non Detected (ND) for control cells. Amplex Red assay was performed following 24 h incubation as a measure of reactive oxygen species (ROS), H2O2. Shown as percentage change from relative control. Boxplot whiskers show median ± Tukey distribution, A; *n* = 6. Student’s T-test was performed for **(A,B)**, ***p < 0.001.

Cells maintained under hypoxic conditions significantly produced styrene as determined by untargeted GG/MS approaches (Figure 3A). Styrene was not found in the headspace of control cells (ND, or not detected) and styrene fluxes in media blanks were not significantly different from zero, while fluxes from hypoxic cells were significantly different from media blanks. Styrene was identified through spectral matching, followed by known standard injections. No other compounds were found to be significantly altered using the untargeted SCAN method.

### 3.4 Reactive oxygen species are reduced under hypoxia

Changes in volatiles, including alkanes, have been linked to increases in ROS (Calenic et al., 2015). The observed uptake of n-Hexane in hypoxic MDA-MB-231 cells could therefore be correlated with alterations in ROS levels in these cells. Following 24 h exposure to hypoxic conditions, ROS, as determined by Amplex Red assay, showed significant reduction compared to control (Figure 3B).

## 4 Discussion

Static headspace sampling chamber was demonstrated to be capable of maintaining a low oxygen environment for >24 h, as evidenced by chamber concentrations and cellular ROS response. Furthermore, VOCs from cells maintained under low oxygen conditions can be sampled, and that these cells produce a significantly different volatile profile than either media blanks or identical cells exposed to hyperoxic conditions.

Two out of 10 compounds targeted by SIM revealed quantifiable, differential metabolic responses in cells exposed to hypoxic conditions (1% O_2_) relative to those maintained in normal laboratory conditions (21% O_2_, physiological hyperoxia). Our previous results quantified alterations in MDA-MD-231 cells for these volatiles after treatment with the chemotherapeutic agent Doxorubicin. When placed under cellular stress through Doxorubicin treatment only MeCl showed a similar stress response (enhanced uptake). In contrast, hexane (or hexane isomers) were not consumed or degraded significantly (Issitt et al., 2022b).

Over 24 h of doxorubicin treatment has been shown to increase ROS (Pilco-FerretoandCalaf, 2016) whereas the opposite has been shown in cells maintained in hypoxic conditions (Sgarbi et al., 2018). A significant reduction was demonstrated in ROS in MDA-MB-231 cells following 24 hs of hypoxia (Figure 3B). Cellular stress response mechanics and differences in cellular state could therefore be identified and quantified through volatile metabolic approaches. Alkanes have been positively correlated with ROS previously (Calenic et al., 2015), here a decrease was demonstrated in n-hexane within hypoxic cells (Figure 2C) with diminished ROS content while in cells treated with doxorubicin, non-significant increases were observed (Issitt et al., 2022b). Metabolic consumption n-hexane is through currently unidentified processes, however the demonstration of variable consumption of a compound demonstrates a potential biomarker dynamics missed by studies only focusing on production. Acetone, hexanes and other compounds shown here are commonly found in urban environments (Redeker et al., 2007) and so their expression in the breath is driven through a combination of equilibration in the bloodstream and chemical/biological uptake processes within the body.

The production of styrene by cells under hypoxia could be a defining VOC biomarker for cancer since hypoxia is characteristic of the tumour microenvironment (SamantaandSemenza, 2018). Our recent review showed that, despite substantial variability in reported outcomes, aromatics are powerful descriptors of cancer (Issitt et al., 2022a). Five studies have previously reported styrene in the breath of lung cancer patients using untargeted approaches (Phillips et al., 1999; Chen et al., 2005a; Peng et al., 2009; Rudnicka et al., 2011; Corradi et al., 2015; Koureas et al., 2020). Styrene has also been reported as higher in the breath of lung cancer patients in studies using other approaches (Chen et al., 2005b; Nardi-Agmon et al., 2016; Wang et al., 2022). However, styrene has been shown to be higher in the breath of smokers (Koureas et al., 2020) and so is often considered, along with other aromatics compounds, to be a confounding contaminant since high percentages of lung cancer patients have a history of smoking. (Issitt et al., 2022a). Styrene has also been reported in the breath of patients with ovarian (Amal et al., 2015), gastric (Amal et al., 2013; Amal et al., 2016) and liver (Qin et al., 2010) cancers.

Styrene utilisation as a breath-based diagnostic biomarker may be challenging since environmental contamination would need to be considered (Hanna et al., 2019). The presented method accounts for environmental VOCs through a flux analysis that incorporates two temporal sampling points, a starting sample following equilibration with the local atmosphere and a second sample at a later time point. This allows us to determine when available environmental volatiles are being added to (metabolically produced) or consumed/degraded by cells. This is important where environmental VOCs may mask effects or differences, such as high traffic, urban environments or perfumed indoor spaces. It is worth stating however, that the observed degradation may be purely non-targeted chemical reactivity with available enzymes or active compounds. However, to some degree whether the process is substrate-specific or nonspecific is unimportant. A different cell response under stress was observed, which points to different cellular states, inclusive of differing enzyme compositions, and points to new and novel potential biomarkers.

Environmental-correction sampling approaches such as this chamber headspace method may present an opportunity to overcome challenges to applications within the clinic, particularly with breath samples taken from ambient air as well as exhalate from the patient. The two time point sampling approach is particularly important since production of compounds with large initial concentrations, or consumption/degradation of compounds are often challenging to detect using single time point sampling methods.

It was observed that cellular consumption of VOCs (MeCl, acetone and n-Hexane) is descriptive of hypoxic stress and that chemotherapeutic stress also induces consumption of VOCs (Issitt et al., 2022b); notably MeCl. To our knowledge this is the first example of a controlled environment experiment performed under low oxygen conditions that both a) quantifies VOC fluxes from a cellular model and b) utilises a VOC injection of gases to monitor ongoing anaerobic metabolism of compounds. We have demonstrated a novel method for induction and maintenance of low oxygen for the study of volatile fluxes. This approach allows new dynamics to be explored for the discovery of cell to patient translational biomarkers. It is perhaps worthy of note that many of the published methods for breath research would not have identified or quantified the methyl chloride or hexane results, due to the small changes (pptv) observed.

It was previously reported that cellular “volatile metabolic flux” can separate cell type and response to chemotherapeutic stress (Issitt et al., 2022b). This chamber-based method has also been successfully used with mice models, quantifying both mouse-breath and faecal volatiles (Issitt et al., 2022b). Here, this chamber-based approach was demonstrated to identify cells under hypoxic stress. A novel method is demonstrated to identify hypoxia-induced VOCs, potential biomarkers of cancer. Importantly these biomarkers are both produced and consumed by cells under hypoxic stress. MeCl, n-hexane and styrene are clinically interesting compounds requiring further investigation. The compounds reported here have been reported as present in human breath (Shahi et al., 2022) and we have shown that these compounds vary in response to cellular stress, from previously published doxorubicin (Issitt et al., 2022b) and here, hypoxic stress. Together this suggests they are able to differentiate cellular response due to pathophysiological differences. These compounds are from diverse functional chemical groups and we have previously demonstrated the ability of functional chemical groups to separate disease groups with greater ability than individually considered compounds (Issitt et al., 2022a). A functionally diverse group of VOCs could give greater power when building a “breath-print” for diagnosis (Issitt et al., 2022a).

## 5 Conclusion

The work presented here demonstrates a novel methodology investigating volatile metabolisms in a controlled environment for volatile biomarker discovery. Using this method we have shown distinct changes in VOCs, demonstrating the potential for VOCs in defining metabolic alterations to environmental changes.

## Data Availability

The raw data supporting the conclusion of this article will be made available by the authors, without undue reservation.

## Supplementary material

SUPPLEMENTARY FIGURE S1 Volatile flux of media controls. Volatile flux in grams per hour (g/hr) for control media (*n* = 8) or media in hypoxia (*n* = 6) in 1Dem dishes. CHCl3, chloroform; OMS, dimethyl sulfide; MeBr, methyl bromide; MeCI, methyl chloride; Mel, methyl iodide; MeSH, methanoethiol; 2-MP, 2 methyl pentane; 3-MP, 3 methyl pentane; n-Hex, n-hexane. Boxplot whiskers show median ± Tukey distribution, *n* = 6. Two way ANOVA followed by Bonferroni post hoc test was performed for **(A,B)**. One way ANOVA with Tukey post-hoc test performed for B; ****p* < 0.001.
