# Supplementary figures and images for: GC/MS analysis of hypoxic volatile metabolic markers in the MDA-MB-231 breast cancer cell line
Source: Front Mol Biosci. 2023 May 11;10:1178269. doi: 10.3389/fmolb.2023.1178269 (PMC10210155; doi:10.3389/fmolb.2023.1178269)

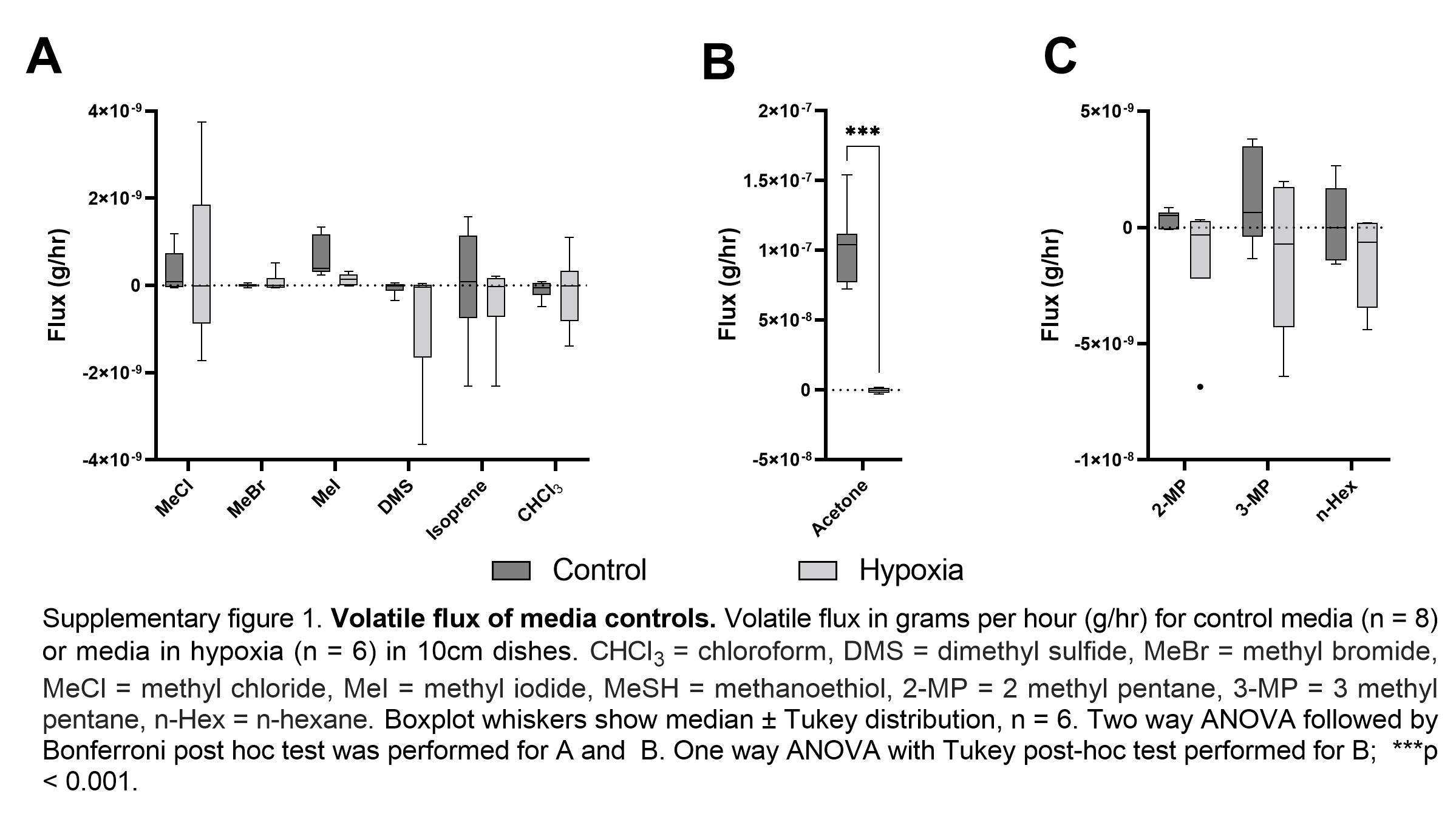

Supplement: Supplementary file 1 [file Image2.jpg]
